# Clinical and Laboratory Parameters Associated with PICU Admission in Children with Multisystem Inflammatory Syndrome Associated with COVID-19 (MIS-C)

**DOI:** 10.3390/jpm14091011

**Published:** 2024-09-23

**Authors:** Maria-Myrto Dourdouna, Evdoxia Mpourazani, Elizabeth-Barbara Tatsi, Chrysanthi Tsirogianni, Charikleia Barbaressou, Nick Dessypris, Athanasios Michos

**Affiliations:** 1 First Department of Pediatrics, Infectious Diseases and Chemotherapy Research Laboratory, Medical School, National and Kapodistrian University of Athens, “Aghia Sophia” Children’s Hospital, 11527 Athens, Greece; myrtodour@med.uoa.gr (M.-M.D.); etatsi@med.uoa.gr (E.-B.T.); 2 Pediatric Intensive Care Unit, “Aghia Sophia” Children’s Hospital, 11527 Athens, Greece; mpourazani.e@hotmail.com (E.M.); chrysanthi.tsirogianni@gmail.com (C.T.); ch.barbar@gmail.com (C.B.); 3 Department of Hygiene, Epidemiology and Medical Statistics, Medical School, National and Kapodistrian University of Athens, 11527 Athens, Greece; ndessyp@med.uoa.gr

**Keywords:** critical care, MIS-C, PICU, SARS-CoV-2, D-dimer, LVEF

## Abstract

**Background/Objectives:** Multisystem Inflammatory Syndrome in children (MIS-C) is a rare but severe post-infectious complication of COVID-19 that often requires admission to the Pediatric Intensive Care Unit (PICU). The present study aimed to compare the demographic, clinical, and laboratory characteristics of children diagnosed with MIS-C who were admitted to the PICU and those who did not require PICU admission. **Methods:** Children diagnosed with MIS-C from September 2020 to April 2023 were included in this case-control study. Demographic, clinical, and laboratory data were collected from medical records. **Results:** Fifty children with MIS-C were included in the study [median (IQR) age: 7.5 (4.3, 11.4) years, 28/50 (56%) males]. Twenty-two (22/50, 44%) children required admission to the PICU. In the multivariate regression analysis, hepatic (OR: 12.89, 95%CI: 1.35–123.41, *p*-value = 0.03) and cardiological involvement (OR: 34.55, 95%CI: 2.2–541.91, *p*-value = 0.01) were significantly associated with hospitalization at the PICU. Regarding the laboratory and imaging parameters during the first 48 h from admission, D-dimer levels higher than 4 μg/mL and decreased Left Ventricular Ejection Fraction (LVEF) were associated with an increased risk of PICU admission (OR: 7.95, 95%CI: 1.48–42.78, *p*-value = 0.02 and OR = 1.28, 95%CI: 1.07–1.53, *p*-value = 0.01). Children who were admitted to the PICU were more likely to develop complications during their hospitalization (10/22, 45.5% vs. 3/28, 10.7%, *p*-value = 0.005) and were hospitalized for more days than children in the pediatric ward (median length of stay (IQR): 20 (15, 28) days vs. 8.5 (6, 14) days, *p*-value < 0.001). **Conclusions:** The findings of this study indicate that cardiovascular and hepatic involvement and increased D-dimer levels in children with MIS-C might be associated with admission to the PICU.

## 1. Introduction

In spring 2020, a case series of pediatric patients presenting with a hyperinflammatory condition that had similarities with Kawasaki Disease (KD) was described as a post-acute immune response to SARS-CoV-2 infection [1,2,3,4]. As more cases were reported globally, the US Centers for Disease Control and Disease Prevention (CDC) and the World Health Organization (WHO) decided to term this clinical entity as Multisystem Inflammatory Syndrome in Children (MIS-C) associated with COVID-19 [5]. MIS-C typically presents 2–6 weeks after infection with SARS-CoV-2, with a wide range of signs and symptoms, including persistent fever, mucocutaneous lesions, gastrointestinal symptoms, and cardiac complications, along with laboratory evidence of systematic inflammation [6,7,8]. 

Although the true global incidence of the syndrome is not clear, MIS-C appears to be a rare sequela of SARS-CoV-2 infection [9,10]. Nevertheless, it is a potentially life-threatening complication of COVID-19, as it could result in severe illness with cardiogenic or distributive shock and multiorgan failure [10,11,12,13]. Notably, a significant portion of children with MIS-C present as critically ill, requiring admission to the Pediatric Intensive Care Unit (PICU), with many of them requiring inotropic support and intubation and, even in some cases, Extracorporeal Membrane Oxygenation [9,14].

While the mortality and the rate of admissions to the PICU in MISC-C are overall higher than in pediatric COVID-19, the long-term effects of the syndrome are not fully elucidated [15,16,17]. Additionally, factors associated with increased MIS-C severity and PICU hospitalization are still not well defined [2,8]. The identification of these factors is essential for the prompt recognition and efficient clinical management of children with severe MIS-C [7]. In the present study, we aimed to compare the demographic, clinical, and laboratory characteristics of children with MIS-C who were admitted to the PICU or not and to identify potential factors associated with an increased risk of admission to the PICU.

## 2. Materials and Methods

### 2.1. Study Design and Participants 

Τhis is a retrospective, case-control study conducted at “Aghia Sophia” Children’s Hospital, Athens, Greece. This facility is the largest tertiary care pediatric hospital in the country, with a capacity of 750 beds. Any child aged 1 month to 16 years old who was admitted to the hospital from September 2020 to April 2023 and had been diagnosed with MIS-C was suitable for inclusion in the study. The study period covered the following periods, which were categorized by the dominant type of SARS-CoV-2 variant during the same time period in Greece: 1 September 2020, to 31 January 2021 (EU1-B.1.177); 1 February 2021, to 31 July 2021 (Alpha variant); 1 August 2021, to 31 December 2021 (Delta variant); and 1 January 2022, to 30 April 2023 (Omicron variant) [18,19].

For the statistical analysis, patients were divided into two groups according to their need for PICU admission: patients admitted to the PICU (PICU group) and patients hospitalized exclusively in the pediatric ward (pediatric ward group). Patients with MIS-C required PICU admission if they met any of the following criteria: (1) Hemodynamic Instability (hypotension, tachycardia for age, oliguria, or anuria), (2) Severe Acute Respiratory Distress, (3) Acute Respiratory Failure (hypoxemia, hypercapnia), (4) Acute Cardiac Failure (acute pulmonary edema), (5) Sepsis-like syndrome.

### 2.2. MIS-C Definition

All the children who were included in the present study met the following CDC 2020 MIS-C case definition criteria: (1) patient’s age <21 years, (2) clinical severe illness requiring hospitalization, (3) no alternative diagnosis, (4) fever (≥1 day), (5) laboratory evidence of inflammation, (6) evidence of SARS-CoV-2 infection or exposure, (7) multisystem (≥2) organ involvement (cardiovascular, renal, respiratory, hematologic, gastrointestinal, mucocutaneous, neurological) [20,21].

### 2.3. Data Collection

Patients’ baseline data including demographics (age, gender) and comorbidities were collected from medical records. Additionally, data regarding COVID-19 including past COVID-19 history, SARS-CoV-2 RT-PCR or antigen test result, and SARS-CoV-2 serology were collected.

Clinical data regarding MIS-C course were recorded. The involvement of each system/organ was recorded based on clinical signs, symptoms, laboratory (peak values), and/or imaging findings. Any clinical signs, symptoms, and/or imaging findings that were consistent with the KD American Heart Association criteria and the Classification Criteria for Macrophage Activation Syndrome (MAS) were also documented [22,23,24].

Additionally, the measurements (during the first 48 h after admission) of the following routinely tested laboratory parameters were obtained from the medical records: Complete Blood Count, biomarkers of inflammation [C-reactive Protein (CRP), Erythrocyte Sedimentation Rate (ESR), Procalcitonin (PCT), and Ferritin] and coagulation [Fibrinogen, International Normalized Ratio (INR), D-dimer levels, Prothrombin Time (PT), and Activated Partial Thromboplastin Time (APTT)], biochemical biomarkers [Aspartate Aminotransferase (AST), Alanine Transaminase (ALT), sodium, and albumin], and cardiac biomarkers, N-terminal prohormone of B-type Natriuretic Peptide (NT-proBNP), and high sensitivity (hs)-Troponin T. Moreover, as cardiac involvement is one of the most prominent features of MIS-C, the first recorded echocardiographic measurement of the Left Ventricular Ejection Fraction (LVEF) was also included in the analysis [25].

Data regarding treatment, management (medications, supportive treatment), and outcomes (PICU admission, days of hospitalization, recovery, complications, mortality) were also collected.

### 2.4. Study Approval 

The study was carried out in accordance with the Declaration of Helsinki and the study protocol was approved by the scientific and bioethics committee of “Aghia Sophia” Children’s Hospital (No. 21736). 

### 2.5. Statistical Analysis

The qualitative parameters were described using absolute (n) and relative (%) frequencies. Comparisons of the qualitative parameters between the PICU group and the pediatric ward group were performed using the Chi-Square test or the Fishers’ Exact test. The quantitative parameters were described using medians (IQR). Comparisons of the quantitative parameters between the PICU group vs. the pediatric ward group were performed using Wilcoxon Rank Sum test. To investigate a possible association between PICU admission and clinical and laboratory parameters, multiple logistic regression analysis was performed and an adjusted Odds Ratio was calculated to express the risk of PICU admission. The statistical analysis was performed with the SAS software V9.4 (SAS Institute Inc., Cary, NC, USA). The level of statistical significance was set at *p*-value < 0.05.

## 3. Results

During the study period, 50 children who fulfilled the MIS-C case definition were identified and included in the present study. Most of the children were admitted to the hospital during the predominance periods of the Delta (16/50, 32%) and Omicron (16/50, 32%) variants. Fourteen out of fifty (28%) children were admitted during the Alpha period and 4/50 (8%) during the EU1-B.1.177 period. 

The clinical and demographic characteristics of the children are outlined in Table 1. The median (IQR) age of the participants was 7.5 (4.3, 11.4) years and 28/50 (56%) children were males. The majority of the children who participated in the study (41/50, 82%) did not have any underlying comorbidities. However, 9/50 (18%) children had (≥1) comorbidities including obesity (5/50, 10%), transfusion-dependent homozygous beta-thalassemia (1/50, 2%), Juvenile Idiopathic Arthritis (JIA) (1/50, 2%), Noonan syndrome (1/50, 2%), postinfectious bronchiolitis obliterans (1/50, 2%), and pulmonary artery stenosis (1/50, 2%). 

A positive RT-PCR test result for SARS-CoV-2 infection on admission was detected in 10/50 (20%) children. SARS-CoV-2 specific antibodies were detected in 39/50 (78%) children. In addition, 31/50 (62%) children had a history of previous SARS-CoV-2 infection and 33/50 (66%) had known previous exposure to SARS-CoV-2.

The most common symptom in children diagnosed with MIS-C was fever (50/50, 100%), followed by gastrointestinal symptoms (43/50, 86%), cardiac involvement (37/50, 74%), and mucocutaneous manifestations (31/50, 62%) (Table 1). The most common treatment administered in our study population was infusion of Intravenous Immune Globulin (IVIG) (49/50, 98%) (Supplementary Table S1). 

Twenty-two out of fifty (44%) participants were admitted to the PICU. During the study period, MIS-C accounted for 3.5% (22/610) of PICU admissions. Τhe main reasons for PICU admission were the following: cardiogenic or vasodilatory shock, respiratory distress, hypoxemic respiratory failure, pulmonary embolism, pulmonary edema, and MAS/Hemophagocytic Lymphohistiocytosis (HLH). 

The univariate comparisons between the clinical and laboratory characteristics of children with MIS-C who were admitted (PICU group) vs. children who were not (pediatric ward group) are presented in Table 1 and Table 2. More specifically, the PICU group had more frequent underlying comorbidities (7/22, 31.8% vs. 2/28, 7.1% *p*-value = 0.03), hepatic involvement (10/22, 45.5% vs. 5/28, 17.9%, *p*-value = 0.03), and cardiovascular involvement (21/22, 95.5% vs. 16/28, 57.1% *p*-value = 0.002) (Table 1). 

Cardiovascular involvement included myocarditis, pericarditis, Coronary Artery Abnormalities (CAA), and Deep Vain Thrombosis and Pulmonary Embolism (DVT-PE). Myocarditis (18/22, 81.8% vs. 14/28, 50%, *p*-value = 0.02) and pericarditis (6/22, 27.3% vs. 1/28, 3.6%, *p*-value = 0.03) were more common in the PICU group vs. the pediatric ward group. The frequencies of Coronary Artery Abnormalities (CAA) and DVT–PE did not differ significantly between the two groups (Table 1 and Supplementary Table S1). LVEF was significantly lower in the PICU group than in the pediatric ward group (*p*-value = 0.001) (Table 1).

Regarding laboratory parameters, the PICU group had significantly higher D-dimer levels (*p*-value = 0.01). Additionally, serum concentrations of hs-Troponin T (*p*-value = 0.01) were higher in the PICU group in comparison with the pediatric ward group (Table 2). 

The multivariate regression model (model 1) regarding clinical parameters associated with PICU admission in MIS-C is presented in Table 3. More specifically, hepatic (OR: 12.89, 95%CI: 1.35–123.41, *p*-value = 0.03) and cardiovascular involvement (OR: 34.55, 95%CI: 2.2–541.91, *p*-value = 0.01) were significantly more common in the PICU group vs. the pediatric ward group (model 1, Table 3). 

In a separate multivariate regression model, regarding the laboratory measurements obtained during the first 48 h from admission and initial LVEF, children with D-dimer levels higher than 4 μg/mL had an increased risk of being admitted to the PICU (OR: 7.95, 95%CI: 1.48–42.78, *p*-value = 0.02) (model 2, Table 3). Additionally, decreased LVEF was associated (OR = 1.28, 95%CI: 1.07–1.53, *p*-value = 0.01) with an increased risk of admission to the PICU (model 2, Table 3).

Concerning management and treatment, statistically significant differences in the PICU group vs. the pediatric ward group were noted in the administration of supplementary oxygen (9/22, 40.9% vs. 0/25, 0%, *p*-value < 0.001), pulse glucocorticoid therapy (11/22, 50% vs. 4/28, 14.3% *p*-value = 0.01), Low Molecular Weight Heparin (16/22, 72.7% vs. 8/28, 28.6%, *p*-value = 0.002), and receptor antagonist of IL-1 (anakinra) (9/22 40.9% vs. 0/28, 0%, *p*-value < 0.001) (Table 1). The patients that received anakinra were diagnosed during the third (1 August 2021 to 31 December 2021) and fourth study subperiods 1 January 2022 to 30 April 2023]. Regarding the patients hospitalized in the PICU, 11/22 (50%) children received inotropes and 11/22 (50%) received vasopressors. Half of the children admitted to the PICU needed hemodynamic support for [median (IQR)] 3 (2, 5) days, and 7/22 (31.8%) children required mechanical ventilation for [median (IQR)] 5 (4, 6) days. Additionally, one child (1/22, 4.5%) received Continuous Renal Replacement Therapy (CRRT) (Supplementary Table S1).

Finally, concerning disease outcomes, the following complications were recorded in our study: persisting myocardial dysfunction, persisting CAA, DVT–PE, pleural effusion, MAS, gastrointestinal bleeding, and acute renal failure requiring CRRT. The development of complications during hospitalization and the persistence of complications at the time of discharge from the hospital were more frequent in the PICU group than in the pediatric ward group (10/22, 45.5% vs. 3/28, 10.7%, *p*-value = 0.005 and 8/22, 36.4% vs. 3/28, 10.7% *p*-value = 0.04, respectively) (Table 1). Furthermore, children in the PICU were hospitalized for more days than children in the pediatric ward [median Length of Stay (LOS) (IQR): 20 (15,28) days vs. 8.5 (6, 14) days, *p*-value < 0.001]. For the PICU group, the median (IQR) LOS in the PICU was 4.5 (3, 7.25) days and the median (IQR) duration of hospitalization at the general pediatric department after PICU discharge was 10.5 (7.75, 18) days. All children were discharged and no deaths were recorded in our study population (Supplementary Table S1).

## 4. Discussion

In the present study, we compared the characteristics of children diagnosed with MIS-C who needed admission to the PICU with those hospitalized in the pediatric ward. Among these 50 patients, various clinical and laboratory parameters associated with PICU admission were identified.

In our setting, a substantial proportion (44%) of the children with MIS-C required admission to the PICU. In other studies, the rates of PICU admission vary from 14% to 80% [2,12,26,27]. This variability may be a result of differences in the diagnostic and management protocols, in the timing of treatment administration, and in the capacity of the PICU of each hospital [2,26]. It could be also attributed to differences in the study periods, as the severity and incidence of the syndrome varies according to the circulating SARS-CοV-2 strain [18,28,29]. Notably, evidence from different studies suggests that the risk and the severity of MIS-C during the Omicron wave were decreased compared to the Alpha or Delta waves [28,29,30].

Regarding baseline demographic parameters, while children requiring PICU admission were older than children hospitalized in the pediatric ward, we did not find any significant association between age and need for PICU hospitalization. By contrast, other studies have reported that older age is associated with PICU admission in MIS-C [2,7,27,31]. In the study by Abrams et al., PICU admission was more likely in children ≥6 years old [7]. Furthermore, in a case series of 183 MIS-C patients, children presenting with shock were significantly older than the children without shock [2,32].

In previous studies, it has been observed that cardiovascular manifestations occur in up to 80% of patients diagnosed with MIS-C [33,34]. Similarly, in our study, cardiovascular involvement was observed in most children (74%) with MIS-C. The cardiac involvement in MIS-C ranges from mild manifestations to severe cardiac complications and includes myocarditis, CAA, arrythmias, pericardial effusion, and shock [35]. The underlying mechanisms of cardiovascular involvement in MIS-C remain elusive and its pathophysiology may be multifactorial [11,36]. Possible mechanisms resulting in cardiovascular complications in MIS-C include the injury to cardiomyocytes from SARS-CoV-2 invasion and microvascular dysfunction and endothelial injury due to the dysregulation of immune responses [11,36]. In our study, cardiovascular manifestations were also more prevalent in children admitted to the PICU (95.5%). Specifically, pericarditis and myocarditis were significantly more common in critically ill children. A prospective study that included 67 MIS-C patients also identified myocarditis as a factor associated with the need for PICU admission [37]. Moreover, another study of 166 MIS-C patients found that hypotension, shock, and myocardial involvement were much more common in children with severe MIS-C [34]. 

Therefore, the measurement of cardiac biomarkers and echocardiographic parameters, like the LVEF, could potentially aid in predicting disease progression in MIS-C [35]. In our study, the concentrations of hs-Troponin T were significantly higher in the PICU group, as described by other studies [7,34]. In contrast with other reported data, in our study, while the first measurements of NT-proBNP were higher in the PICU group, there was not a significant difference between the two groups [7]. In the multivariate model for quantitative parameters, lower LVEF values were identified as a potential risk factor for PICU admission. Decreased LVEF has been associated with severe disease courses in MIS-C in other studies [35,38,39]. Beaver et al. reported that lower LVEF at admission was associated with the need for vasoactive medication and Tran et al. reported that LVEF (<60%) was associated with the risk of developing shock and PICU admission in MIS-C patients [35,38]. These findings underline the importance of close echocardiographic monitoring of children with MIS-C.

According to our findings, during the MIS-C course, hepatic involvement (peak liver enzymes and/or imaging findings) was significantly more common in the PICU group. Although in our study the levels of liver enzymes (AST or ALT) at admission did not differ significantly between the two groups of patients, in a case series of MIS-C patients, an elevation of AST at presentation was reported in patients that required critical care compared to those that did not [40]. Furthermore, in line with our findings, a multi-center study of MIS-C patients also found that hepatomegaly was associated with PICU admission [34]. The involvement of the liver during the course of MIS-C could be a result of an immune-mediated underlying mechanism [41]. In MIS-C, the immense release of pro-inflammatory cytokines leads to organ dysfunction, including liver damage [41]. Hence, the higher frequency of hepatic involvement in the PICU group may be secondary to the shock and the more profound organ dysfunction that is observed in these patients [40].

Regarding laboratory findings, children diagnosed with MIS-C who had D-dimer levels above a specific threshold (higher than 4 μg/mL) at presentation had an increased risk of being admitted to the PICU. The role of D-dimers has already been underscored in MIS-C, as evidence suggests that the syndrome is characterized by a prothrombotic inflammatory state [39,42,43]. An association between D-dimer levels and PICU admission has also been observed in other studies [8,34,44]. In children with severe COVID-19, including MIS-C, D-dimer levels were associated with a higher risk not only of PICU admission, but also of intubation, myocardial dysfunction, and development of sequelae [42]. As D-dimer measurements are easy to obtain through routine blood testing, serial measurements should be included in the routine laboratory evaluation of children with MIS-C [42].

Other laboratory markers obtained in the acute phase of MIS-C have been associated with PICU admission, like elevated ESR (>30 mm/h), CRP (>5 mg/dL), ferritin, PCT, thrombocytopenia, lymphopenia, hyponatremia, and decreased serum albumin levels [8,26,27,31,35,45,46]. Significant differences in the above biomarkers were not detected in our population, possibly due to the limited number of participants. Still, altogether, the above findings emphasize the importance of obtaining repeated laboratory measurements in these children [7].

Concerning treatment, a subset of children did not respond to the initial infusion of IVIG and needed a second infusion of IVIG (30%), pulsed glucocorticoids (30%), or administration of IL-1 receptor antagonist (anakinra) (18%). The administration of pulsed glucocorticoids and anakinra was more common in the PICU group. 

A significant number of children developed complications during their hospitalization, with complications being more prevalent in critically ill children. In our study population, CAA were detected in 10% of the children. In accordance with our findings, the prevalence of CAA in MIS-C is estimated at approximately 8–26% [47,48]. Τhe incidence of MAS/HLH in our study was lower (4%) than that reported in previous studies (18–76%) [34]. Markedly, two children presented with clinical features similar to acute appendicitis. Similar cases have been reported in the literature and suggest that MIS-C could either mimic the disease or present together with complicated forms of acute appendicitis [49].

Finally, in our study, all children were discharged from hospital and no deaths were recorded. Other studies have also reported a rather low mortality rate in MIS-C, estimated at approximately 1.9% [50,51]. As it has been described in other reports, in a subset of the children, especially in those with critical illness, complications including residual myocardial dysfunction did not fully resolve at the time of discharge [51]. However, in a three-month multidisciplinary follow-up, these complications resolved, suggesting that MIS-C has an overall favorable outcome in children who had received appropriate treatments.

Our study has several limitations. The major limitation of this study is the relatively small number of participants. Due to the small sample size, it was not possible to perform sub-analysis by age groups and we may have not been able to detect significant differences in some of the clinical features that were less frequently observed in our study groups. Also, although we tried to perform the most adequate analysis concerning the small number of participants, some of the results can be only indicative and may not be precise. However, MIS-C appears to be a rare complication of SARS-CoV-2 infection, with an initially estimated incidence of 45–54 cases/100.000 infected children <15 years old [52]. Also, given that its incidence has further declined during the Omicron wave, we believe that data from similar series are important [52]. Additionally, in our study, we have included children diagnosed with MIS-C during the predominance periods of several different SARS-CoV-2 variants (EU1-B.1.177, Alpha, Delta, Omicron) in Greece. Moreover, although our center is the largest tertiary care pediatric hospital in the country, another limitation of the present study is its single-center study design. The single-center nature of our study and the relatively small sample size limit the generalizability of our findings, as the study may not fully reflect different patient populations and healthcare settings. Moreover, another limitation of our study is that we did not examine how evolving MIS-C treatments during the study period might have affected the risk of PICU admission.

## 5. Conclusions

The findings of our study indicate that in patients with MIS-C, cardiovascular and hepatic involvement might be associated with hospitalization in the PICU. In addition, D-dimer levels above a specific threshold could possibly aid in predicting which child with MIS-C will require admission to the PICU, while decreased LVEF was recognized as a potential risk factor for PICU admission. Although the generalizability of our study results is limited by the relatively small number of participants, the above findings highlight the importance of specific laboratory and echocardiographic measurements in children with MIS-C for the early identification of high-risk patients. 

## Figures and Tables

**Table 1:** Demographic characteristics and clinical features of 50 children diagnosed with MIS-C and hospitalized from September 2020 to April 2023, at “Aghia Sophia” Children’s Hospital, Athens, Greece.

	Total Nο of Participants	PICU Admission	Pediatric Ward Hospitalization	*p*-Value
**Total Study Population**	50 (100)	22 (44)	28 (56)	n/a
**Demographic Characteristics**				
Age (years) *	7.5 [4.3, 11.4]	9.1 [4.4, 11.4]	7.2 [4.3, 11]	0.74 ^a^
**Gender (Males)**	28 (56)	12 (54.6)	16 (57.1)	0.85 ^b^
**Comorbidities**	9 (18)	7 (31.8)	2 (7.1)	**0.03** ^c^
**Clinical Presentation**				
**Fever**	50 (100)	22 (100)	28 (100)	n/a
**Mucocutaneous manifestations**	31 (62)	13 (59.1)	18 (64.3)	0.71 ^b^
**Cervical Lymphadenitis**	12 (24)	5 (22.7)	7 (25)	0.85 ^b^
**Gastrointestinal Involvement I** (Abdominal pain, vomiting, diarrhea)	43 (86)	19 (86.4)	24 (85.7)	>0.99 ^c^
**Gastrointestinal Involvement II** (Hepatic Involvement)	15 (30)	10 (45.5)	5 (17.9)	**0.03** ^b^
**Respiratory Involvement**	12 (24)	7 (31.8)	5 (17.9)	0.25 ^b^
**Kidney Involvement**	18 (36)	11 (50)	7 (25)	0.07 ^b^
**Central Nervous System Involvement**	5 (10)	3 (13.6)	2 (7.1)	0.64 ^c^
**Cardiovascular Involvement**	37 (74)	21(95.5)	16 (57.1)	**0.002** ^b^
LVEF (%) *	65 [60, 70]	60 [55, 65]	68 [65, 71]	**0.001** ^a^
**Myocarditis**	32 (64)	18 (81.8)	14 (50)	**0.02** ^b^
**Pericarditis**	7 (14)	6 (27.3)	1 (3.6)	**0.03** ^c^
**Coronary Artery Abnormalities**	5 (10)	4 (18.2)	1 (3.6)	0.16 ^b^

Notes: Values are referred to as absolute frequencies (relative frequencies, %) or * Median [IQR]. *p*-value obtained after: ^a^ Wilcoxon rank sum test, ^b^ Chi-Square, ^c^ Fisher’s exact test. Statistically significant differences (*p*-value < 0.05) are marked in bold. Abbreviations: MIS-C: Multisystem inflammatory syndrome in children associated with COVID-19, PICU: Pediatric Intensive Care Unit, n/a: nonapplicable, LVEF: Left Ventricular Ejection Fraction.

**Table 2:** Laboratory data of 50 children diagnosed with MIS-C and hospitalized from September 2020 to April 2023, at “Aghia Sophia” Children’s Hospital, Athens, Greece.

Laboratory Parameter	Total Nο of Participants	PICU Admission	Pediatric Ward Hospitalization	*p*-Value
**Complete Blood Count**				
Hgb (g/dL)	11.1 [9.8, 12.1]	11 [9.9, 12]	11.4 [9.6, 12.5]	0.42
WBC (×10^3^/μL)	9.95 [7.2, 15.3]	9.7 [7.3, 13.9]	10 [7.1, 15.8]	0.69
Neutrophils (%)	81.8 [69, 87]	83.4 [74.2, 87]	78.8 [65, 86]	0.33
Lymphocytes (%)	8.9 [6.5, 23.1]	7.4 [6, 15.4]	12.2 [6.9, 23.5]	0.23
Platelets (×10^3^/μL)	190 [157, 352.5]	187.5 [150, 385]	193 [167, 350]	0.72
**Inflammation Biomarkers**				
ESR (mm/hr)	70 [32, 95]	65 [25, 80]	79 [35, 100]	0.16
CRP (mg/L)	135 [88.7, 230]	137 [89.7, 229]	129 [87.6, 231]	0.99
PCT (μg/L)	2.29 [1, 7.7]	3.1 [1, 8.6]	2.2 [0.8, 7.6]	0.56
Ferritin (μg/L)	460 [314, 989]	460 [306, 989]	502.5 [329, 1055]	0.84
**Coagulation Biomarkers**				
D-dimer (μg/mL)	3.1 [2, 6]	4.8 [2.7, 6.9]	2.4 [1.5, 4]	**0.01**
INR	1.3 [1.2, 1.4]	1.3 [1.2, 1.5]	1.3 [1.2, 1.4]	0.49
Fibrinogen (μg/mL)	498 [407, 603]	441 [386, 610]	500 [421, 603]	0.52
PT (sec)	15.1 [13.5, 16.4]	15.1 [14, 17.3]	15 [13.3, 16.1]	0.39
APTT (sec)	32.6 [29.8, 36.4]	34.3 [30, 37.2]	31.9 [29.5, 35.4]	0.30
**Cardiac Biomarkers**				
hs-Troponin T (pg/mL)	8.2 [5.1, 20.2]	11.8 [7.2, 44.8]	5.6 [3.8, 14.6]	**0.01**
NT Pro-BNP (pg/mL)	1987.5 [371.8, 4479.5]	3419 [320.5, 7262]	1727.5 [694, 3295]	0.33
**Biochemical Biomarkers**				
AST (IU/L)	27 [20, 49]	29 [20, 53]	24 [21, 36]	0.88
ALT(IU/L)	23 [14, 41,5]	26.5 [13, 42]	22 [15, 34]	0.95
Albumin (g/dL)	3.7 [3.5, 4.2]	3.7 [3.5, 3.9]	3.8 [3.5, 4.3]	0.18
Na (mmol/L)	135 [132.5, 136]	134.5 [132, 136]	136 [133, 136]	0.13

Notes: Values are reported as Median [IQR], *p*-values were obtained after Wilcoxon rank sum test. Statistically significant differences (*p*-value < 0.05) are marked in bold. Abbreviations: MIS-C: Multisystem inflammatory syndrome in children associated with COVID-19, PICU: Pediatric Intensive Care Unit, Hgb: Hemoglobulin, WBC: White Blood Cells, ESR: Erythrocyte Sedimentation Rate, CRP: C-Reactive Protein, PCT: Procalcitonin, INR: International Normalized Ratio, PT: Prothrombin Time, APTT: Activated Partial Thromboplastin Time, hs-Troponin Τ: high sensitivity Troponin T, NT Pro-BNP: N-terminal prohormone of B-type Natriuretic Peptide, AST: Aspartate Aminotransferase ALT: Alanine Transaminase.

**Table 3:** Multiple logistic derived Odds Ratios (OR) with 95% Confidence Intervals (95%CI) for admission to the PICU by clinical (model 1) and laboratory and echocardiographic measurements at presentation (model 2) of children diagnosed with MIS-C.

Variable	Category or Increment	OR	95%CI		*p*-Value
**Model 1 (Clinical parameters)**					
Comorbidities	Yes vs. no	7.52	0.83	67.80	0.07
Hepatic Involvement	Yes vs. no	12.89	1.35	123.41	**0.03**
Kidney Involvement	Yes vs. no	1.60	0.34	7.49	0.55
Cardiovascular Involvement	Yes vs. no	34.55	2.20	541.91	**0.01**
**Model 2 (Laboratory and echocardiographic measurements at presentation)**					
D-dimer	4+ vs. 4 μg/mL	7.95	1.48	42.78	**0.02**
hs-Troponin T	One (pg/mL) more	1.00	0.99	1.02	0.76
LVEF	One % less	1.28	1.07	1.53	**0.01**

Abbreviations: PICU: Pediatric Intensive Care Unit, MIS-C: Multisystem inflammatory syndrome in children associated with COVID-19, hs-Troponin Τ: high sensitivity Troponin T, LVEF: Left Ventricular Ejection Fraction.

## Data Availability

The data that support the findings of this study are available from the corresponding author (A.M.) upon reasonable request.

## Supplementary Materials

The following supporting information can be downloaded at: https://www.mdpi.com/article/10.3390/jpm14091011/s1, Table S1: Treatment and disease outcomes of 50 children diagnosed with MIS-C and hospitalized at “Aghia Sophia” Children’s Hospital, Athens, Greece.
