# Supplementary material for: Clinical and Laboratory Parameters Associated with PICU Admission in Children with Multisystem Inflammatory Syndrome Associated with COVID-19 (MIS-C)
Source: J Pers Med. 2024 Sep 23;14(9):1011. doi: 10.3390/jpm14091011 (PMC11432765; doi:10.3390/jpm14091011)
Supplement: Supplementary file 1 [file jpm-14-01011-s001.zip › jpm-3205270-supplementary.pdf]

Supplementary Table S1. Treatment and disease outcomes of 50 children diagnosed with MIS-C and hospitalized at "Aghia Sophia" Children's Hospital, Athens, Greece.

	Total No of participants	PICU Admission	Pediatric Ward Hospitalization	*p*-value
**Total Study Population**	50 (100)	22 (44)	28 (56)	n/a
	**Treatment**			
**IVIG**	49 (98)	22 (100)	27 (96.4)	>0.99 [a]
**IVIG Second Infusion**	15 (30)	9 (40.9)	6 (21.4)	0.14 [b]
**Glucocorticoid therapy**	48 (96)	20 (90.9)	28 (100)	0.19 [a]
Glucocorticoid therapy 2mg/kg	47 (94)	19 (86.4)	28 (100)	0.08 [a]
Glucocorticoid Pulses	15 (30)	11 (50)	4 (14.3)	**0.01 [b]**
**Aspirin**	21 (42)	6 (27.3)	15 (53.6)	0.06 [b]
**LMWH**	24 (48)	16 (72.7)	8 (28.6)	**0.002 [b]**
**IL-1 receptor antagonist (anakinra)**	9 (18)	9 (40.9)	0 (0)	**<0.001 [a]**
**Antibiotics**	47 (94)	22 (100)	25 (89.3)	0.25 [a]
**Supplemental Oxygen**	9 (19.1)	9 (40.9)	0 (0)	**<0.001 [a]**
**Other treatments**	18 (36)	15 (68.2)	3 (10.7)	**<0.001 [b]**
	**Outcomes**			
**Complications (≥1)**	13 (26)	10 (45.5)	3 (10.7)	**0.005 [b]**
Persistence of Myocardial Dysfunction	3 (6)	2 (9.1)	1 (3.6)	0.58 [a]
Persistence of Coronary Artery Abnormalities	3 (6)	3 (13.6)	0 (0)	0.08 [a]
Pulmonary Embolism/Deep Vein Thrombosis	2 (4)	1 (4.6)	1 (3.6)	>0.99 [a]
Pleural Effusion	2 (4)	1 (4.5)	1 (3.6)	>0.99 [a]
MAS/HLH	2 (4)	2 (9.1)	0 (0)	0.19 [a]
Gastrointestinal bleeding	1 (2)	1 (4.5)	0 (0)	0.44 [a]
Acute Kidney Injury requiring CRRT	1 (2)	1 (4.5)	0 (0)	0.44 [a]
**Hospital LOS***	15 [7, 20]	20 [15, 28]	8.5 [6, 14]	**<0.001 [c]**
**Residual complications at discharge**	11 (22)	8 (36.4)	3 (10.7)	**0.04 [a]**
**Discharge**	50 (100)	22 (44)	28 (56)	n/a
**Mortality**	0 (0)	0 (0)	0 (0)	n/a

Notes: Values are referred to as absolute frequencies (relative frequencies, %) or * Median [IQR]. p-value obtained after: [a] Fisher's exact test, [b] Chi-Square, [c] Wilcoxon rank sum test.

Abbreviations: MIS-C; Multisystem inflammatory syndrome in children associated with COVID-19, PICU; Pediatric Intensive Care Unit, n/a; non applicable, IVIG; Intravenous Immune globulin, LMWH; Low Molecular Weight Heparin, IL-1; Interleukin-1, MAS/HLH; Macrophage activation syndrome (MAS) and Hemophagocytic Lymphohistiocytosis (HLH), CRRT; Continuous Renal Replacement Therapy, Hospital LOS; Length of Stay.
